# Restricting carbohydrates and calories in the treatment of type 2 diabetes: a systematic review of the effectiveness of ‘low-carbohydrate’ interventions with differing energy levels

**DOI:** 10.1017/jns.2021.67

**Published:** 2021-09-14

**Authors:** Anna P. Nicholas, Adrian Soto-Mota, Helen Lambert, Adam L. Collins

**Affiliations:** 1 Department of Nutritional Sciences, University of Surrey, Guildford, UK; 2 Department of Physiology, Anatomy & Genetics, University of Oxford, Oxford, UK

**Keywords:** Diabetes, Low-carbohydrate diet, Low-energy diet, Obesity, HbA1c, glycated haemoglobin, LCD, low-carbohydrate diets, LED, low-energy diets, T2D, type 2 diabetes, TDR, total diet replacement

## Abstract

There are two proven dietary approaches to shift type 2 diabetes (T2D) into remission: low-energy diets (LEDs) and low-carbohydrate diets (LCDs). These approaches differ in their rationale and application yet both involve carbohydrate restriction, either as an explicit goal or as a consequence of reducing overall energy intake. The aims of this systematic review were to identify, characterise and compare existing clinical trials that utilised ‘low-carbohydrate’ interventions with differing energy intakes. Electronic databases CENTRAL, CINAHL, Embase, MEDLINE and Scopus were searched to identify controlled clinical trials in adults with T2D involving low-carbohydrate intake (defined as <130 g carbohydrate/d) and reporting weight and glycaemic outcomes. The initial database search yielded 809 results, of which fifteen studies met the inclusion criteria. Nine out of fifteen studies utilised LCDs with moderate or unrestricted energy intake. Six trials utilised LEDs (<1200 kcal/d), with all except one incorporating meal replacements as part of a commercial weight loss programme. Interventions using both restricted and unrestricted (*ad libitum*) energy intakes produced clinically significant weight loss and reduction in glycated haemoglobin (HbA1c) at study endpoints. Trials that restricted energy intake were not superior to those that allowed *ad libitum* low-carbohydrate feeding at 12 and 24 months. An association was observed across studies between average weight loss and reduction in HbA1c at 6, 12 and 24 months, indicating that sustained weight loss is key to T2D remission. Further research is needed to specifically ascertain the weight-independent effects of carbohydrate restriction on glycaemic control in T2D.

## Graphical abstract

**Figure:**
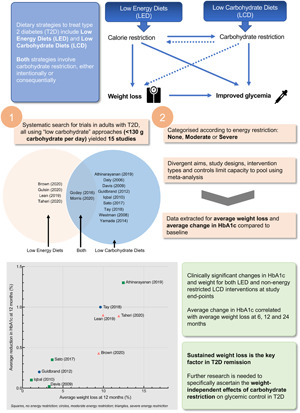

## Introduction

The prevalence of type 2 diabetes (T2D) has reached epidemic proportions and is a major global concern. According to the World Health Organization (WHO), more than 422 million people have diabetes worldwide, representing a global prevalence among adults of 8⋅5 %^(1)^. In the UK alone, over 3⋅9 million people are diagnosed with diabetes, 90 % of which have T2D, and this figure is anticipated to rise to more than 5 million by 2025^(2)^. T2D is a major risk factor for other health conditions including cardiovascular disease, kidney failure, neuropathy and blindness^(1)^. It has also recently emerged as a significant risk factor for COVID-19^(3)^.

T2D used to be considered a chronic progressive disease typically managed by escalating pharmacotherapy to maintain normoglycaemia and mitigate disease complications. However, the paradigm of treatment is changing with recognition that T2D can be put into remission^(4)^. Remission seems to occur up to a point, beyond which the pancreatic β-cells are unable to recover^(5)^. Definitions for remission vary but it is generally defined as achieving glycaemia below the diabetic range in the absence of antiglycaemic medications for at least 1 year^(4)^. The American Diabetes Association (ADA) defines three states of remission: (1) ‘partial’ – subdiabetic hyperglycaemia [glycated haemoglobin (HbA1c) 5⋅7–6⋅4 %] for at least 1 year; (2) ‘complete’ – normoglycaemic (HbA1c level <5⋅7 %) for at least 1 year; (3) ‘prolonged’ – complete remission for at least 5 years^(6)^.

**Fig. 1.**
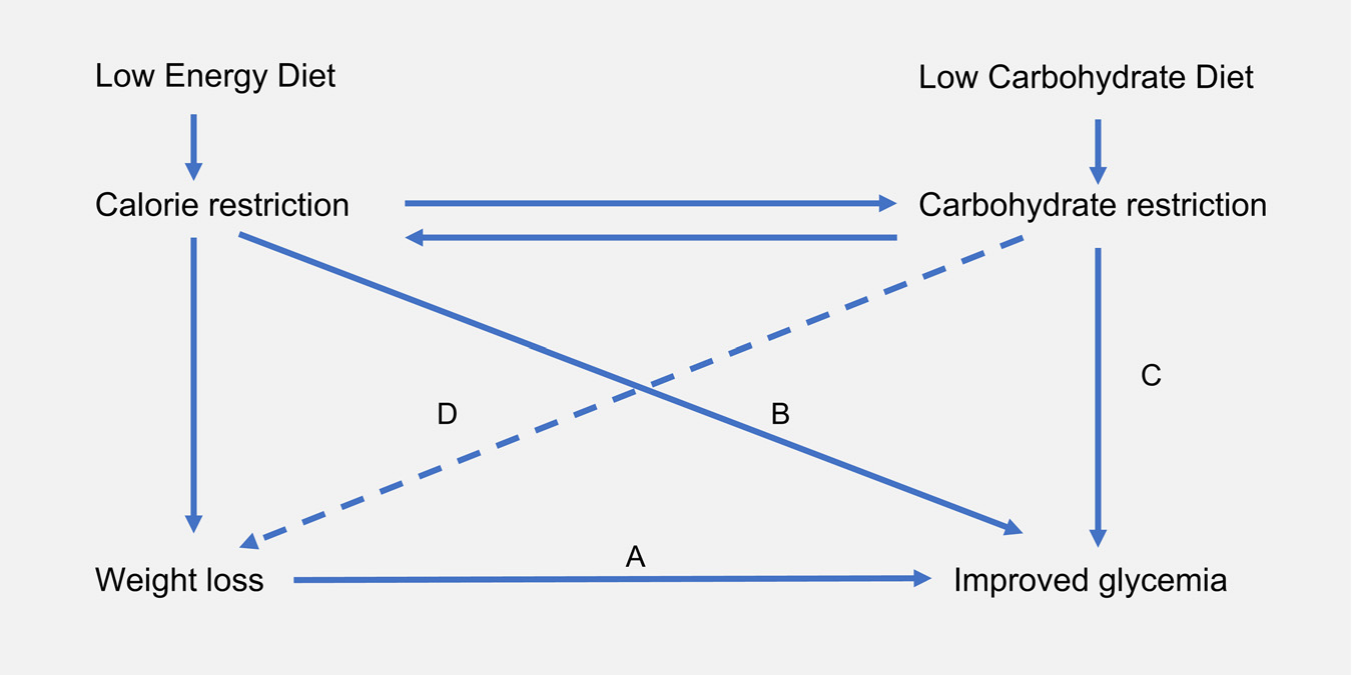
Interrelationship between energy restriction, weight loss and carbohydrate restriction in improved glycaemic control: carbohydrate and energy restriction are interrelated. (A) In obese individuals with T2D, weight loss is associated with improved glycaemic control^(10)^. This is in accordance with the twin cycle hypothesis, whose central tenet is that excess lipids within the liver and the pancreas drive T2D pathogenesis^(11)^. (B) In studies of low-energy feeding, glycaemia improves within days of energy restriction, before significant weight loss has occurred^(12)^. (C) Carbohydrate restriction improves glycaemia by reducing postprandial glucose rises. While failed repression of gluconeogenesis and glycogenolysis are major causes of hyperglyacemia^(13)^, dietary carbohydrate intake is the largest driver of postprandial glucose rises. (D) Carbohydrate restriction is also associated with weight loss. This may occur as a function of spontaneous energy restriction or there may be independent effects arising from reduced insulin secretion. Whether or not carbohydrate restriction has independent effects on body weight remains a matter of contentious debate (hence depicted as dashed line)^(14,15)^. T2D, type 2 diabetes.

There are currently two proven non-surgical ways to achieve T2D remission: low-energy diets (LEDs) and low-carbohydrate diets (LCDs)^(7–9)^. These two approaches focus on operating different metabolic levers: energy restriction and carbohydrate restriction. Given that both factors are interlinked (Fig. 1), it is not clear which is driving T2D remission and hence which offers the most effective interventional approach.

LEDs restrict energy intake to induce rapid weight loss^(16)^. They characteristically provide between 800 and 1200 kcal/d utilising either total diet replacement (TDR) or some inclusion of conventional foods as a partial diet replacement^(17)^. LEDs have gained attention for their use in diabetes management after the 2011 Counterpoint study which demonstrated the normalisation of β-cell function, hepatic insulin sensitivity and fasting blood glucose using an 8 week 600 kcal/d diet^(18)^. In 2018, the DiRECT trial demonstrated that an intensive weight management programme using TDR could achieve T2D remission in 46 % of participants after 1 year^(8)^.

LCDs specifically aim to restrict carbohydrate intake, either as a percentage of total energy (TE) or as an absolute intake (g/d). Definitions of what constitutes ‘low carbohydrate’ have been inconsistent over time and between studies but the definitions proposed by Feinman *et al.*^(19)^ are becoming more widely accepted. Specifically, this defines low carbohydrate as <26 % of TE from carbohydrates or 130 g/d and very low-carbohydrate ketogenic diets as <10 % of TE from carbohydrates or 50 g/d.

Table 1 highlights the similarities and differences between LCDs and LEDs. Despite their differences, carbohydrate restriction is common in both: LCDs restrict carbohydrates as an explicit goal, whereas LEDs restrict carbohydrates as a consequence of achieving low-energy intake.

**Table 1.** Generalised similarities and differences between low carbohydrate diets and low energy diets

	Low-energy diets	Low-carbohydrate diets
**Meal format**	Usually meal replacement soups, shakes and bars	Usually food-based
**Energy restriction**	Energy restricted to ≤1200 kcal/d	Variable. Some allow *ad libitum* feeding to satiety; others include moderate energy restriction (>1200 kcal/d)
**Carbohydrate restriction**	Variable carbohydrate content but usually at least 50 g CHO/d	Restricted to <130 g CHO/d; VLCKD restricted to 20–50 g CHO/d
**Duration**	Necessarily restricted to short periods of up to 3–5 months	No limit on duration

CHO, carbohydrate; VLCKD, very low-carbohydrate ketogenic diet.

There is currently much interest in the use of carbohydrate restriction to treat T2D^(20)^. Over the past 5 years, ten meta-analyses, based on nearly fifty randomised controlled trials (RCTs), have aimed to address the question of whether diets low in carbohydrates produce greater improvements in weight and glycaemic control compared with higher carbohydrate control diets^(21–30)^. The majority of these meta-analyses have found a beneficial effect from carbohydrate restriction^(21–27)^, and none have favoured higher carbohydrate comparators, although several studies have found no difference between diets^(28–30)^. Most recently, Goldenberg *et al.*^(27)^ performed a comprehensive meta-analysis of the effect of LCD (<130 g/d) on T2D remission. They identified higher rates of diabetes remission among LCDs compared with low-fat diets at 6 months, an effect which diminished at 12 months. The authors highlighted the potential confounding role of calorie restriction but found no evidence of credible effect modification when intervention and control diets were calorically matched compared with when they were not. However, this could have been due to measurement error since calorie intakes were determined by dietary questionnaires.

Thus, the role of energy restriction in LCDs remains unclear, and no study to date has attempted to group together both LCDs and LEDs that are also ‘low carbohydrate’ in absolute terms. This review takes an alternative approach to the existing evidence base by recognising the commonality between these two approaches ‘clamped’ by carbohydrate intake. Specifically, it aims to review, characterise and compare the clinical trials that have used low-carbohydrate (<130 g/d) approaches with different levels of energy intake.

## Methods

### Data sources and searches

The present systematic review was performed with reference to the Cochrane Handbook for Systematic Reviews of Interventions^(31)^ and reported in accordance with the Preferred Reporting Items for Systematic Reviews and Meta-Analyses (PRISMA) statement^(32)^. A protocol was registered with PROSPERO in advance (CRD42020197257)^(33)^.

An electronic search was performed using the databases Medline, EMBASE, CINAHL, Scopus and Cochrane Central Register of Controlled Trials (CENTRAL). The search was performed on 7 July 2020, and no date limits were applied. Search terms included keywords and subject headings related to T2D, LEDs or LCDs, glycaemic outcomes and clinical trials (see Supplementary material). A manual search of reference lists of key systematic reviews and reports was also conducted to identify any additional relevant studies. Search results were independently reviewed by A.P.N. and A.S.M. and any conflicts over inclusion were resolved by discussion.

### Study selection

Studies were eligible for inclusion if they were controlled trials including adults diagnosed with T2D, involving an LCD (defined as <130 g/d or <26 % of TE) and reporting a change in weight and glycaemic outcomes (including HbA1c, fructosamine, fasting plasma glucose and/or glycaemic variability). Non-randomised trials were eligible to allow the inclusion of trials in more ecologically valid settings, such as those utilising very low-energy weight-loss diets. Control diets that stipulated any other type of dietary intervention such as low fat, ‘healthy eating’ and Mediterranean, or usual diabetes care were permitted. All countries and languages were eligible. For full inclusion and exclusion criteria, see Table 2.

**Table 2.** Study inclusion and exclusion criteria

Inclusion criteria	Exclusion criteria
RCTs and CCTs using low CHO diets or very low-energy diets with <130 g/d or <26% TE from CHO in adults (≥18 years) with T2D Control is any other type of dietary intervention or usual care Data from crossover trials with washout periods of ≥4 weeks between interventions. In the absence of adequate washout period, data from these trials only included if able to extract relevant data for the first phase Studies of individuals with and without T2D only if subgroup analysis available for participants with T2D Studies reporting change in weight and markers of glucose control Provided macronutrient goals/CHO goals as TE or g/d	Studies that included people with other chronic diseases (except hypertension or CVD) or taking systemic corticosteroids, or had any progressive disease requiring hospital care Studies involving participants undergoing surgery Studies of T1D, prediabetes or gestational diabetes Studies with enteral or parenteral feeds Participants aged <18 years or pregnant/lactating women Treatment diet poorly defined; unclear whether low CHO criteria were met Studies involving intermittent fasting protocols Study duration <1 week Studies based on medication co-intervention not applied to all groups

RCTs, randomised controlled trials; CCTs, controlled clinical trials; CHO, carbohydrate; TE, total energy; T2D, type 2 diabetes; CVD, cardiovascular disease; T1D, type 1 diabetes.

### Data extraction and quality assessment

Data were extracted by A.P.N. Data items included: study characteristics, participant characteristics, details of the intervention (including prescribed and reported energy and macronutrient composition), dietary adherence, medication changes and outcome data for HbA1c and weight loss at 3, 6, 12 and 24 months (where available) and study endpoints. HbA1c only was collected as it is the most widely used marker of T2D remission, and quality of life outcomes were not reported due to lack of consistency across studies. These represent minor deviations from the protocol submitted to PROSPERO. The mean percentage weight loss from baseline and the mean absolute reduction in HbA1c were calculated for intervention arms. Absolute rather than relative change in HbA1c was used, since therapeutic goals are based on a threshold value and not relative reduction^(34)^. Risk of bias was assessed using the Cochrane Risk of Bias tool^(35)^.

### Data synthesis and analysis

A narrative synthesis was undertaken to explore the characteristics of the included studies. HbA1c and weight loss outcome data were compared between intervention and control arms within studies at the longest duration time point.

To compare weight and HbA1c outcomes between studies, percentage weight loss and HbA1c change at study endpoint and at specific time points (3, 6, 12 and 24 months) were plotted graphically in scatter plots. Meta-analysis was not considered appropriate due to high clinical heterogeneity between studies. The association between average weight loss and HbA1c change was examined using correlation analysis and the computation of *R*^2^ values in Prism 8 for OS X Version 8.4.3^(36)^.

## Results

### Search results

**Fig. 2.**
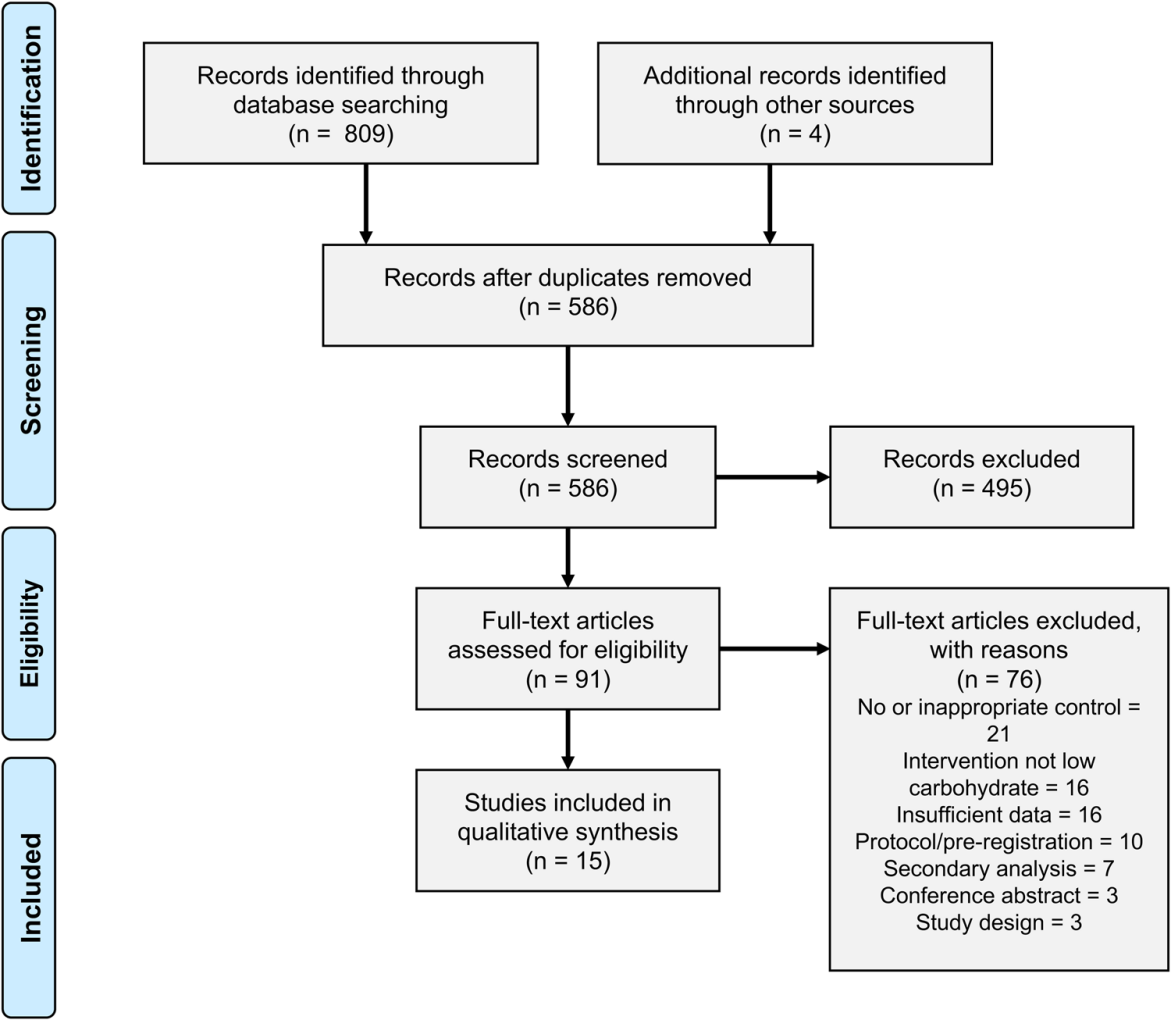
Study screening and selection.

Fig. 2 shows the selection of studies, in accordance with the PRISMA guidelines^(32)^. The initial database search yielded 809 studies, of which 223 were duplicates. Following title and abstract screening, ninety-one studies were retrieved for full-text screening. A total of fifteen studies met the inclusion criteria.

### Study characteristics

This review yielded a highly heterogeneous set of studies that fulfilled criteria for ‘low carbohydrate’. The characteristics of the fifteen controlled trials are summarised in Table 3. The publication period covered from 2006 to 2020, study duration ranged from 3 to 24 months and study sample sizes ranged from 12 to 262 participants in the intervention arm. Of the included studies, thirteen were RCTs and two were non-RCT. Of the two non-RCTs, one was randomised at the level of primary care practice rather than the participant level.

**Table 3.** Characteristics of included studies

Study	Location, setting	Sample size (intervention/ total)	Length (months)	Objectives	Primary outcome (s)	Intervention	Control	Prescribed carbohydrate intake (g/d)	Prescribed energy intake (kcal/d)
Unrestricted energy intake (*ad libitum*)									
Athinarayanan *et al*.^(37)^	USA, app-based with or without onsite-clinic support	262/349	24	To assess the effectiveness and safety of a novel digitally monitored continuous care intervention including nutritional ketosis at 2 years	Retention, HbA1c, HOMA-IR, fasting glucose, fasting insulin, c-peptide and weight	Metabolic and continuous care intervention + nutritional ketosis	Usual diabetes care	<30	Unrestricted (instructed to achieve satiety without tracking energy intake)
Daly *et al*.^(38)^	UK, Hospital outpatient	51/102	3	To examine effects of a 3 month programme of dietary advice to restrict carbohydrate intake compared with reduced-portion, low-fat advice in adults with obesity and poorly controlled T2D	Change in weight, HbA1c, TC:HDL, TAG	Low carbohydrate	Low fat	<70	Unrestricted (although energy-balance principles incorporated into education)
Davis *et al*.^(39)^	USA, Research centre	55/105	12	To compare effects of a 1 year intervention with a low-carbohydrate and a low-fat diet on weight loss and glycaemic control in adults with T2D	Weight, HbA1c	Low carbohydrate	Low fat	20–25 (increase by 5 g/week)	Unrestricted
Iqbal *et al*.^(40)^	USA, Hospital outpatient	70/144	24	To determine whether comparable results to those of short-term, intensive interventions comparing a low-carbohydrate versus low-fat diet in adults with obesity and T2D could be achieved over 24 months using a low-intensity intervention that approximates what is feasible in outpatient practice	Weight, HbA1c, glucose and lipids	Low carbohydrate	Low fat	30	Unrestricted
Sato *et al*.^(41)^	Japan, Hospital outpatient	33/66	6	To compare effectiveness and safety of low-carbohydrate diet with energy-restricted diet	HbA1c	Low carbohydrate	Energy-restricted	130	Unrestricted
Westman *et al*.^(42)^	USA, Outpatient research centre	38/84	6	To test whether a diet lower in carbohydrates would lead to greater improvement in glycaemic control over 24 weeks in obese adults with T2D	HbA1c	Low-carbohydrate ketogenic	Low GI, reduced energy	<20	Unrestricted
Yamada *et al*.^(43)^	Japan, Outpatient clinic	12/24	6	To examine effects of a non-energy-restricted, low-carbohydrate diet in Japanese patients unable to adhere to an energy-restricted diet	HbA1c	Low carbohydrate	Conventional energy-restricted	70–130	Unrestricted
Moderate energy restriction (1200–2000 kcal/d)									
Guldbrand *et al*.^(44)^	Sweden, Primary care	30/61	24	To compare effects of a 2 year intervention with a low-fat diet or a low-carbohydrate diet based on four group meetings to achieve compliance	Weight, HbA1c	Low carbohydrate	Low fat	85	1800 men/1600 women
Tay *et al*.^(45)^	Australia, Research centre	58/115	24	To compare effects of a very low-carbohydrate, high unsaturated fat, low saturated fat diet with a high carbohydrate, low-fat diet on glycaemic control and CVD risk factors in T2D	HbA1c	Low-carbohydrate, high unsaturated fat, low saturated fat	High carbohydrate, low fat	<50	Individualised to achieve 500–1000 kcal deficit per day
Severe energy restriction (<1200 kcal/d)									
Brown *et al*.^(46)^	UK, Secondary care outpatient	45/90	12	To examine the effect of a low-energy TDR intervention *v.* standardised dietetic care in patients with long-standing T2D and obesity receiving insulin therapy	Weight	12 week low-energy TDR by Cambridge Weight Plan, followed by structured food reintroduction and weight loss maintenance programme	Standardized dietetic care on weight loss (600 kcal deficit diet)	115	800–820
Goday *et al*.^(47)^	Spain, Hospital outpatient	45/89	4	To evaluate the short-term safety and tolerability of a VLCK diet (≤50 g of carbohydrate daily) in an interventional weight loss programme including lifestyle and behavioural modification support (Diaprokal Method) in subjects with T2D	Safety parameters including renal function, liver function and plasma uric acid, Na and K	Very low-energy- ketogenic diet involving three phases (Pronokal Method)	Low-energy diet	<50	600–800
Gulsin *et al*.^(48)^	UK, Research centre	29/90	3	To confirm the presence of subclinical cardiovascular dysfunction in working-age adults with T2D and determine whether this is improved by a low-energy meal replacement diet or exercise training	Measure of diastolic function: change in left ventricular peak early diastolic strain rate	12 week low-energy TDR by Cambridge Weight Plan	Two other arms: routine care as per NICE guidelines and a supervised aerobic exercise programme	101	810
Lean *et al*.^(49)^	UK, Primary care	149/306	24	To assess whether intensive weight management within routine primary care would achieve remission of T2D	Weight loss of 15 kg or more and remission of diabetes	12 week low-energy total TDR by Cambridge Weight Plan, followed by food reintroduction and weight loss maintenance programme	Usual diabetes care	124	825–853
Morris *et al*.^(50)^	UK, Primary care	21/48	3	To examine the feasibility of a food-based, low-energy, low-carbohydrate diet with behavioural support delivered by practice nurses for patients with T2D	Prespecified feasibility criteria to progress to a full trial	Food-based, low-energy, low-carbohydrate diet	Usual diabetes care	<59	800–1000
Taheri *et al*.^(51)^	Qatar, Primary care	70/158	12	To assess whether an intensive lifestyle intervention would lead to significant weight loss and improved glycaemia in young individuals with early diabetes	Weight loss	12 week low-energy total TDR by Cambridge Weight Plan, followed by food reintroduction and weight loss maintenance programme	Usual diabetes care	124	825–853

HbA1c, glycated haemoglobin; HOMA-IR, Homeostatic Model Assessment of Insulin Resistance; T2D, type 2 diabetes; TC, total cholesterol; HDL, high-density lipoprotein; TAG, blood triacylglycerol; GI, glycaemic index; CVD, cardiovascular disease; TDR, total diet replacement; VLCK, very low-calorie ketogenic; NICE, The National Institute for Health and Care Excellence.

### Variable approaches to energy and carbohydrate restriction

**Fig. 3.**
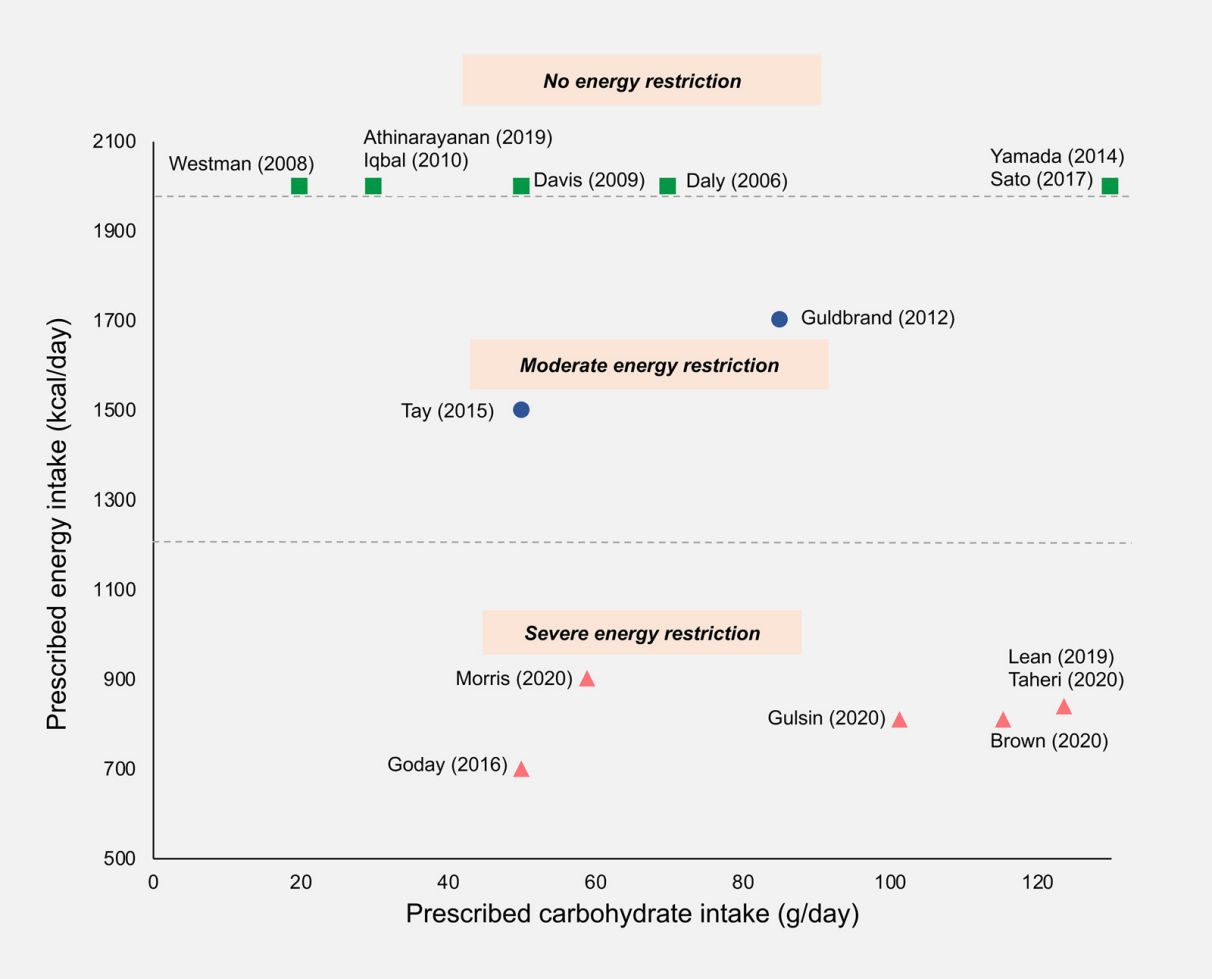
Prescribed daily carbohydrate and energy intakes in included studies**.** Where a maximum allowance of carbohydrate or energy was prescribed, this value was used; where a range of carbohydrate or energy intakes was prescribed, the mid-point value was taken; where energy intake was unrestricted, a value of 2000 kcal/d was assigned. Squares, no energy restriction (*ad libitum* feeding); circles, moderate energy restriction (1200–2000 kcal/d); triangles, severe energy restriction (<1200 kcal/d).

Studies were categorised into three groups based on their prescribed energy intakes: unrestricted (*ad libitum* feeding), moderately restricted (1200–2000 kcal/d) or severely restricted (<1200 kcal/d) (Fig. 3).

A total of seven out of fifteen studies involved traditional low or very LCDs that were food-based and did not prescribe limits on energy intake^(37–43)^. Two out of fifteen studies used moderate energy reduction targets^(44,45)^. Almost all of the studies in these groups aimed for sustained carbohydrate restriction throughout the study period. Only two were initiated by a very restrictive early phase followed by subsequent increases in carbohydrate allowance^(38,45)^.

Six out of the fifteen trials restricted energy intake to <1000 kcal/d^(46–51)^, with all except one incorporating meal replacements as part of a stepped weight loss programme. Three studies used a 3–5 month proprietary TDR weight loss phase involving energy restriction of around 800 kcal/d, followed by food reintroduction and weight loss maintenance phases^(46,49,51)^. In the TDR phase, carbohydrates accounted for 50–59 % of TE which translated to 100–125 g/d. The TDR phase was low carbohydrate in absolute terms but the macronutrient composition of subsequent phases was unclear from the published reports. These studies did not explicitly prescribe carbohydrate restriction but rather aimed to achieve weight loss via energy restriction. There were two exceptions to this: Morris *et al.*^(50)^ used a food-based diet that was explicitly low carbohydrate (<26 % of TE) as well as low energy (800–1000 kcal/d)^(45)^ and Goday *et al.*^(47)^ used a very low-energy ketogenic diet. All of the LED trials were recently published, allowing only a limited period for follow-up.

### Study aims

The principle aim for the majority of energy-restricted studies was to achieve and maintain weight loss, which was accomplished using mixed interventions with a number of different strategies including meal replacements to achieve <1200 kcal/d, individualised dietary advice, lifestyle changes and medication, if necessary^(52)^. For these studies, the control group was a variant of usual care. By contrast, the principal aim of most of the LCD studies was to test the effect of manipulating carbohydrate intake on participants’ weight and glycaemic control, with a more conventional ‘low-fat’ diet as a comparator.

### Outcome measures

Most studies’ primary outcome measures related to weight and/or glycaemic control with the exception of three studies assessed cardiac function, safety parameters and full-trial feasibility criteria. All studies reported HbA1c, with nine out of fifteen studies specifying HbA1c as a primary outcome. Four studies, all published in or after 2018, reported T2D ‘remission’ or ‘reversal’, although definitions varied^(37,46,49,51)^.

Twelve out of fifteen trials reported medication changes although methods of reporting were heterogeneous (Supplementary Table S5). All of the twelve studies that included medication changes reported a greater reduction in diabetic medications in the intervention compared with the control group. Six out of fifteen studies attempted to assess the quality of life using a range of questionnaires (Supplementary Table S1).

### Baseline participant characteristics

Mean population ages ranged from 42 to 69 years, and there was a mix of ethnicities and gender ratios across studies. All studies except those conducted in Japan recruited participants who had a BMI of >30 kg/m^2^, with the majority having a mean BMI of >35 kg/m^2^. There was a wide range of average diabetes duration (2–14 years) and medication usage (Supplementary Table S2).

### Intervention details

Interventions varied across studies in several ways including mode of delivery, dietary advice provided, intensity of support and utilisation of behavioural strategies to promote adherence (Supplementary Table S3). Some studies included very low-intensity interventions (involving only infrequent group sessions and dietary advice), whereas others involved more intensive input and employed a range of strategies to support dietary and lifestyle change including behaviour change techniques, intensive group support, biomarker feedback and health technologies, online support and remote care. More recent studies, published in or after 2017, involved higher intensity mixed interventions, typically employing a range of technological and behavioural support.

### Control

Six out of fifteen studies used usual care as a control. In five of these studies, usual care provided minimal input, so the intervention and control arms differed in a number of aspects beyond dietary change. The remaining nine studies all used a version of a low-fat, energy-restricted diet. In all but one study, energy intake was not matched between intervention and control diets. The exception was Tay *et al.*^(45)^ who used a planned isocaloric control, advising both arms to limit energy intake to achieve a 500–1000 kcal deficit per day.

### Dietary assessment and adherence

The majority of studies attempted to assess dietary adherence in some way, although the methods employed varied between studies. A total of eight studies used food records (1, 3, 5 or 7-d food diaries) and two studies used self-report via questionnaires. Dietary adherence was not assessed in any of the four studies that utilised TDR (via meal replacements). Three studies used urinary or blood ketones as a marker of nutritional ketosis, one of which reported adherence based on these data^(37)^. Of note, in those that included participant reported carbohydrate intakes, seven out of eight studies had reported values that exceeded the prescribed carbohydrate intake by more than 10 % (Supplementary Table S4).

### Risk of bias

**Fig. 4.**
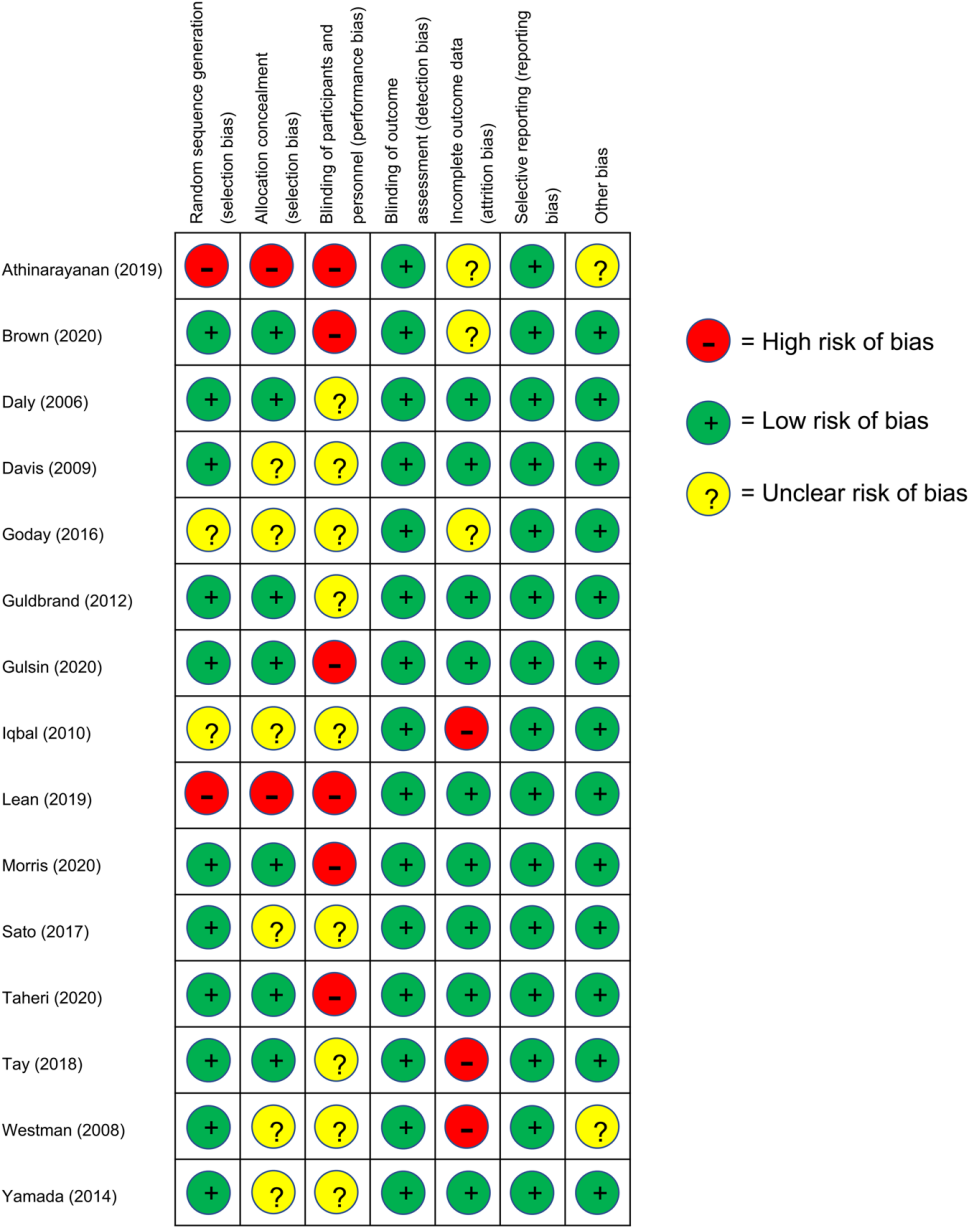
Risk of bias assessment results: +, low risk of bias; ?, unclear risk of bias; –, high risk of bias.

Selection bias was high for the two studies in which participants self-selected into the intervention; it was low or unclear for the thirteen trials that randomised participants between intervention arms. Six studies provided insufficient information on allocation concealment. Performance bias was judged to be high in the six studies which involved mixed interventions that differed in several aspects to the control arm, due to the nature of these trials. Performance bias was unclear for the remainder of trials due to the possible influence of participant or personnel expectations on the results. Detection bias was considered low for all studies based on the objective nature of the outcomes of interest. Attrition bias was high in three studies in which dropout rates were high or imbalanced between groups and completer analysis was used. Reporting bias of the outcomes of interest was low in all studies, since pre-specified outcomes of interest were reported. Only two studies were judged as unclear for ‘other bias’, due to potential baseline imbalances in confounders between the groups. The risk of bias assessment is summarised in Fig. 4.

### Effectiveness of interventions

#### Between-group differences

All but one study resulted in a reduction in HbA1c between baseline and study endpoint (Table 4). A total of eight studies demonstrated significant improvements in HbA1c in the intervention arm compared with the comparator arm. All studies reported weight loss from baseline to endpoint, with nine studies showing greater weight loss in the intervention arm compared with the control group. All studies using usual diabetes care as a control arm reported significant between-group differences in weight and HbA1c. Only two of the five studies reporting data at 24 months found a difference between intervention and control groups by the end of the study^(37,49)^.

**Table 4.** Baseline and change values for HbA1c and weight for intervention and control arms at longest available time-point

Author (date)	Duration (months)	HbA1c (%)							Weight						
		Intervention			Control			Between-group difference	Intervention			Control			Between-group difference
		Baseline mean	sd	Change	Baseline mean	sd	Change		Baseline mean	sd	Change (kg, %)	Baseline mean	sd	Change (kg, %)	
Unrestricted energy intake (*ad libitum*)															
Athinarayanan (2019)	24	7⋅6	1⋅5*	−0⋅9	7⋅6	1⋅8*	0⋅4	Sig.	114⋅6	0⋅6*	−11⋅9, −10⋅4	112⋅4	1⋅9*	1⋅3, 1⋅1	NS
Daly (2006)	3	9⋅0	0⋅2	−0⋅6	9⋅1	0⋅2	−0⋅2	NS	101⋅6	1⋅8	−3⋅6, −3⋅5	102⋅3	2⋅5	0⋅9, 0⋅9	NS
Davis (2009)	12	7⋅5	1⋅5	0⋅0	7⋅4	1⋅5	0⋅2	NS	93⋅6	18⋅0	−3⋅1, −3⋅3	101⋅0	19⋅0	−3⋅1, −3⋅1	NS
Iqbal (2010)	24	7⋅9	1⋅7	−0⋅1	7⋅6	1⋅3	−0⋅2	NS	118⋅3	21⋅3	−1⋅5, −1⋅3	115⋅5	16⋅7	−0⋅2, 0⋅2	NS
Sato (2017)	6	8^†^	*NR*	−0⋅7	8⋅3^†^	*NR*	0	Sig.	74^†^	*NR*	−1⋅6, −2⋅2	73⋅6^†^	*NR*	−0⋅6, −0⋅8	Sig.
Westman (2008)	6	8⋅8	1⋅8	−1⋅5	8⋅3	1⋅9	−0⋅5	Sig.	108⋅4	20⋅5	−11⋅1, −10⋅2	105⋅2	19⋅8	−6⋅9, −6⋅6	Sig.
Yamada (2014)	6	7⋅6	0⋅4	−0⋅6	7⋅7	0⋅6	−0⋅2	Sig.	67⋅0	15⋅9	−2⋅6, −3⋅9	68⋅1	7⋅7	−1⋅4, −2⋅1	NS
Moderate energy restriction (1200–2000 kcal/d)															
Guldbrand (2012)	24	7⋅5	3⋅1	0⋅0	7⋅2	2⋅9	0⋅0	NS	88⋅0	16	−2⋅3, −2⋅2	90⋅6	19⋅0	−3⋅0, −3⋅3	NS
Tay (2018)	24	NR	NR	−0⋅6	NR	NR	−0⋅9	NS	101⋅7	NR	−6⋅8, −6⋅7	101⋅6	NR	−6⋅6, −6⋅5	NS
Severe energy restriction (<1200 kcal/d)															
Brown (2020)	12	8⋅8	1⋅7	−0⋅4	9⋅3	1⋅7	−0⋅1	NS	104⋅0	20⋅2	−9⋅8, −9⋅4	103⋅1	18⋅9	−5⋅6, −5⋅6	Sig.
Goday (2016)	4	6⋅9	1⋅1	−0⋅9	6⋅8	1⋅0	−0⋅4	Sig.	91⋅5	11⋅4	−14⋅7, −16⋅1	90⋅0	11⋅3	−5⋅1, −5⋅6	Sig.
Gulsin (2020)	3	7⋅2	1⋅1	−1⋅0	7⋅3	0⋅9	−0⋅1	NR	106⋅7	16⋅2	−13⋅6, −13⋅6	102⋅6	14⋅9	−1⋅1, −1⋅0	NR
Lean (2019)	24	7⋅7	1⋅3	−0⋅5	7⋅5	1⋅1	0⋅0	Sig.	101⋅0	16⋅7	−7⋅6, −7⋅5	98⋅8	16⋅1	−2⋅3, −2⋅3	Sig.
Morris (2020)	3	7⋅9	1⋅4	−1⋅5	7⋅4	0⋅8	−0⋅1	Sig.	103⋅0	16⋅7	−9⋅5, −9⋅2	97⋅6	13⋅2	−2⋅0, −2⋅0	Sig.
Taheri (2020)	12	7⋅0	1⋅4	−0⋅9	7⋅0	1⋅2	−0⋅4	Sig.	100⋅6	17⋅0	−12⋅0, −11⋅9	101⋅7	19⋅2	−4⋅0, −3⋅9	Sig.

HbA1c, glycated haemoglobin; sd, standard deviation; Sig., significant; NS, non-significant; NR, not reported.

Data taken for longest available time point. All data are mean and standard deviation unless specified.

* Standard error of the mean, sem.

† Median and interquartile range.

#### Weight loss and HbA1c change in intervention arms

**Fig. 5.**
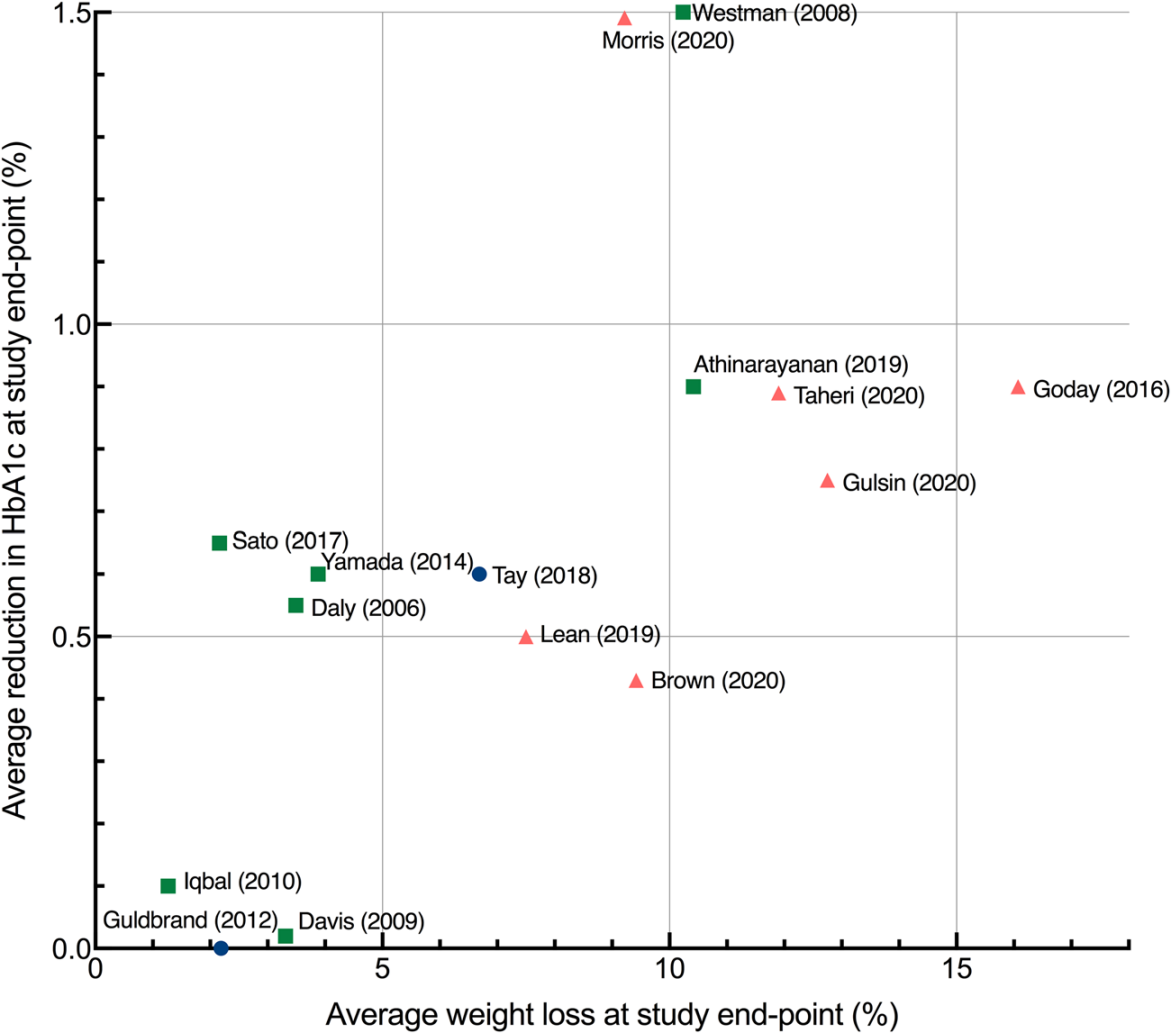
Average improvement in HbA1c and average percentage weight loss at study endpoints. Each point represents the mean value for a single study with the exception of Sato *et al.*^(41)^ which represents median values. Study endpoints range from 3 to 24 months. Squares, no energy restriction (*ad libitum* feeding); circles, moderate energy restriction (1200–2000 kcal/d); triangles, severe energy restriction (<1200 kcal/d).

There was a wide range of reported improvements in the intervention arms across studies in mean HbA1c change (ranging from 0⋅0 to 1⋅5 %) and mean percentage weight loss (ranging from only 1 to over 15 % of baseline weight). Fig. 5 shows the data for all study endpoints.

Those trials that severely restricted energy all produced clinically significant weight loss of more than 5 %, whereas energy-unrestricted trials produced a wider range of weight and HbA1c outcomes. The non-energy-restricted studies were more numerous, published over a longer time period and involved more diverse intervention types. Two of the most effective interventions explicitly combined low-carbohydrate and low-energy approaches^(47,50)^.

**Fig. 6.**
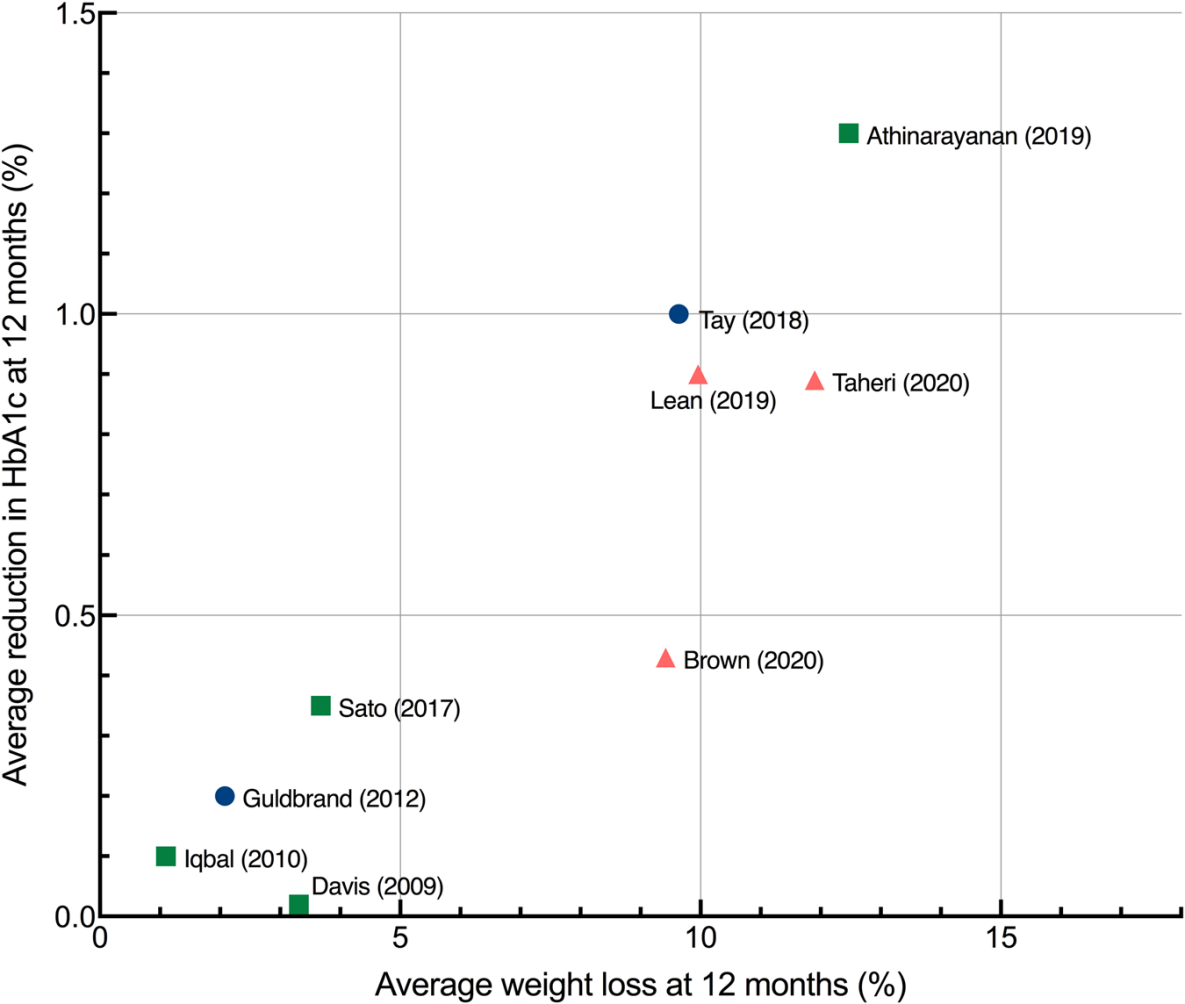
Average improvement in HbA1c and average percentage weight loss at 12 months each point represents the mean changes from baseline in HbA1c and weight for a single study, with the exception of Sato *et al.*^(41)^ which represents median values. Studies were only included if they reported data at 12 months. Squares, no energy restriction (*ad libitum* feeding); circles, moderate energy restriction (1200–2000 kcal/d); triangles, severe energy restriction (<1200 kcal/d). HbA1c, haemoglobin A1C.

Fig. 6 shows the data at 12 months to facilitate comparisons between studies. The level of energy restriction did not clearly distinguish intervention efficacy. The three studies using LEDs led to a consistent and considerable mean weight loss of around 10 %^(17,49,51)^. The two studies reporting the largest changes at both 12 and 24 months involved LCDs with unrestricted or moderate energy restriction^(37,45)^. The most effective intervention at 12 and 24 months involved an *ad libitum* ketogenic diet^(37)^.

#### Association between weight loss and HbA1c

An association was observed between average weight loss and change in HbA1c across studies at 6, 12 and 24 months. The association was strongest at longer study lengths, as assessed by *R*^2^: at 6 and 12 months, 84 and 82 %, respectively, of the variation in average HbA1c change between studies could be accounted for by the variation in average weight loss; at 24 months, this increased to 91 %. A scatterplot summarises the results at 12 months (Fig. 6). Reductions in HbA1c were associated with the increased percentage of weight loss.

## Discussion

This systematic review took a novel approach to the clinical trial evidence regarding dietary approaches to treat T2D by recognising that carbohydrate restriction is a common feature of LCDs and LEDs. Previous systematic reviews with meta-analyses have assessed the impact of higher *v.* lower carbohydrate diets^(21–30)^. These have shown either no effect^(28–30)^ or a positive effect of carbohydrate restriction on weight loss and HbA1c^(21–27)^ and have noted the role of spontaneous energy restriction in LCDs as a potential confounder.

A key strength of this review is that it only included low-carbohydrate studies that adhered to the definitions outlined by Feinman *et al.*^(19)^. Previous systematic reviews have often included studies with higher thresholds of carbohydrate intake which limits understanding of the effects of ‘true’ low-carbohydrates diets.

### Risk of bias

The heterogeneity of the study designs gave rise to different risks of bias when studies were evaluated with the Cochrane Risk of Bias tool. Studies that aimed to assess the efficacy of mixed interventions (involving dietary, physical, and behavioural changes) were judged as high risk of performance bias, as they may document larger effects than those only assessing dietary changes. Additionally, two studies involved patients self-selecting the treatments they underwent which, although a valid approach for assessing efficacy, may also bias them towards reporting larger effects^(37,49)^. The heterogeneity of the (mostly design-inherent) sources of bias precludes head-to-head comparisons.

### Intervention efficacy

This review found a range of intervention effectiveness that was not clearly distinguished by the level of energy restriction: both energy-restricted and energy-unrestricted diets were effective at 12 months, and the most effective intervention at 12 and 24 months involved an *ad libitum* energy-unrestricted diet^(37)^. This reinforces others’ observations of spontaneous energy restriction in LCDs^(53)^ and highlights the potential efficacy of both low-carbohydrate and low-energy intervention types in the treatment of T2D.

The strength of the association between average weight loss and HbA1c change at 6, 12 and 24 months was notable. This finding is consistent with the ‘Twin Cycle Hypothesis’ of T2D which proposes that T2D can be put into remission following weight loss, which reverses the accumulation of fat in the pancreatic β-cells, thereby restoring their function^(11)^. The potential causal relationship between weight loss and diabetes remission remains a matter of investigation^(54,55)^.

Regardless of causality, the strength of the association between weight loss and glycaemic markers underscores the importance of interventions that can maintain weight loss in the longer term. Weight maintenance is the most challenging area of weight management. Low-energy meal replacement-based diets are capable of producing dramatic weight loss^(56)^ but they are necessarily short term and weight regain is common upon cessation, especially in the absence of continued support^(57)^. DiRECT^(49)^ was the only LED trial to report data beyond 12 months and it will be of interest to see if the results achieved can be sustained over the full 5-year trial period. This review identified a greater number of clinical trials testing LCDs or very LCDs, and a correspondingly wider range of outcomes. As with DiRECT, it will be of interest to see if the results using an *ad libitum* ketogenic diet in the study by Athinarayanan *et al.*^(37)^ can be maintained over the full 5-year trial period.

In line with this focus on weight loss maintenance, this review identified a trend towards interventions with greater levels of participant support through co-interventions (involving exercise, pharmacotherapy, sleep and stress-reduction), new technologies and behaviour change techniques. Previous research shows that, regardless of the modality of weight loss, participant support is important^(58)^, and this represents a promising trend in research.

### Independent role of carbohydrate restriction

It is not clear from this review if carbohydrate restriction directly affects T2D status independent of weight loss. None of the included studies robustly measured energy intake or used an isoenergetic control, meaning the influence of spontaneous energy restriction was not controlled or accounted for. Tay *et al.*^(45)^ included a planned energy-matched high-carbohydrate control but the diet was undertaken in a free-living environment and participants in the low-carbohydrate arm reported lower energy intakes than those in the low-fat arm. Several short-term studies do indicate a weight-independent effect of carbohydrate restriction on glycaemic control^(59–61)^ and there are other plausible underlying mechanisms that remain under investigation^(62,63)^.

The field would greatly benefit from further research to explore the potential for an independent effect of carbohydrate restriction on glycaemic control. This could be tested using a parallel-arm clinical trial comparing low-energy meal replacements with varying proportions of carbohydrates across a large enough range. Trials similar to this have been conducted using low-energy formula diets with 100 g (40 %) *v.* 162⋅5 g (65 %) carbohydrates per day and 1000 kcal for 4 weeks^(60)^ and <40 g *v.* 65–156 g/d for 3 weeks each (in a crossover trial)^(59)^. These trials have found that manipulating carbohydrates leads to differences in various markers of metabolic health. Trials using a broader range of carbohydrate intakes at fixed energy levels are needed to further explore these findings.

### Implications for clinical practice

The data in this review indicate that a major factor in T2D remission is weight loss maintenance. In clinical practice, patients would benefit from receiving information about the available options to enable them to make a fully informed individual choice, and to select for the diet and lifestyle changes that they can adhere to over the longer term, which may or may not incorporate carbohydrate restriction.

## Limitations

There are several limitations in the current literature and in this review. First, presenting average weight and HbA1c outcomes of studies did not account for the underlying individual variability in weight and HbA1c outcomes.

Secondly, intervention efficacy was based solely on weight and HbA1c change. Some studies reported outcomes including sleep quality, anxiety and quality of life, as well as other glycaemic outcomes such as fasting blood glucose and glycaemic variability. There is also growing use and application of continuous glucose monitoring which provides measures of short-term glycaemic control such as time in target range^(64,65)^. Future reviews could consider the inclusion of these and other outcomes to provide a more holistic review of the effectiveness of LEDs and LCDs in the treatment of T2D.

Thirdly, inconsistent reporting of medication adjustment across studies meant that changes in HbA1c were not considered in the context of medication changes. This may have masked differences in effect size between interventions and led to an underestimation of the positive impact of carbohydrate restriction on glycaemic control^(22,24,25,27,29)^. Future clinical trials would benefit from a more standardised approach to reporting medication changes to facilitate comparisons between studies.

Fourthly, due to heterogeneity in dietary assessment methods and inaccuracies associated with self-reported intakes^(66)^, carbohydrate and energy quantities were based on prescribed rather than actual intakes. Diet studies often suffer from poor adherence to the prescribed diet^(67)^ and this review also found that reported carbohydrate intake exceeded prescribed carbohydrate intake in the majority of studies. Conclusions are therefore limited to the dietary prescription of carbohydrate restriction, rather than carbohydrate restriction *per se*.

Finally, this review did not distinguish between ketogenic and non-ketogenic diets. Ketones have been shown to directly lower hyperglycaemia by suppressing hepatic glucose output^(68,69)^. However, the role of ketosis in long-term weight loss is contentious due in part to poor adherence rates to ketogenic diets in some clinical trials^(40)^. This is reflected in a recent systematic review that found that LCDs were more effective than very low-carbohydrate ketogenic ones, an effect which diminished when adherence was accounted for^(27)^.

## Conclusions

This review took a novel approach to the dietary strategies for T2D remission by recognising the commonality of carbohydrate restriction between LEDs and LCDs. It found that trials that severely restricted energy intake were not superior to those that allowed *ad libitum* low-carbohydrate feeding (no prescribed energy deficit) at longer study durations (12 and 24 months). However, the strong association between average weight loss and HbA1c change at 6, 12 and 24 months indicates that successful interventions for T2D are those that enable sustained weight loss in the longer term. Further studies that carefully match carbohydrate and/or energy intake between arms are needed to establish the independent roles of carbohydrate and energy restriction in T2D treatment.

## Supplementary material

For supplementary material accompanying this paper visit http://dx.doi.org/10.1017/jns.2021.67. click here to view supplementary material
