# Supplementary material for: Restricting carbohydrates and calories in the treatment of type 2 diabetes: a systematic review of the effectiveness of ‘low-carbohydrate’ interventions with differing energy levels
Source: J Nutr Sci. 2021 Sep 14;10:e76. doi: 10.1017/jns.2021.67 (PMC8453456; doi:10.1017/jns.2021.67)
Supplement: Supplementary file 1 [file S2048679021000677sup001.docx]

Supplementary Material

Search strategy

In accordance with PRISMA guidelines, below is the search string used for CENTRAL.

"Type 2 Diabet*" OR "T2D" OR "T2DM" OR "Non insulin dependent diabet*" OR "Non-insulin-dependent diabet*" OR "Noninsulin dependent diabet*" OR "Noninsulin-dependent diabet*" OR "Non-insulin dependent diabet*" OR "NIDDM" OR "Stable Diabet*" OR "Type II diabet*" OR "Type Two diabet*" OR "Maturity-Onset Diabet*" OR "Maturity Onset Diabet*" OR "Slow-Onset Diabet*" OR "Slow Onset Diabet*" OR "Adult-Onset Diabet*" OR "Adult Onset Diabet*" OR "diabetes mellitus type 2" OR "diabetes mellitus type II" OR "dm type 2" OR "dm type II" in Title Abstract Keyword AND "low carbohydrate" OR "low-carbohydrate" OR "carbohydrate restricted" OR "carbohydrate-restricted" OR "low carbohydrate high fat" OR "LCHF" OR "low CHO" OR "low-CHO" OR "ketogenic" OR "VLCKD" OR "carbohydrate reduced" OR "carbohydrate-reduced" OR "nutritional ketosis" OR "low calorie" OR "low-calorie" OR "low energy" OR "low-energy" OR "calorie restrict*" OR "calorie-restrict*" OR "caloric restrict*" OR "VLED" OR "VLCD" OR "energy restrict*" OR "hypocaloric" OR "diet replacement" OR "meal replacement" OR "restrict* calorie" OR "restrict* caloric" OR "liquid formula diet" OR "low-calorie liquid diet" OR "low caloric" in Title Abstract Keyword AND "HbA1" OR "Hb A1c" OR "HbA1c" OR "Glycosylated Hemoglobin" OR "Glycosylated Haemoglobin" OR "Glycated Hemoglobin" OR "Glycated Haemoglobin" OR "Glycohemoglobin A" OR "Hemoglobin A" OR "Haemoglobin A" OR "glucose variability" OR "time in range" OR "time in target" OR "continuous glucose monitor" OR "CGM" OR "fructosamine" OR "blood glucose" OR "blood serum glucose" OR "blood sugar" OR "normoglycaemia" OR "normoglycemia" OR "plasma glucose" OR "postprandial glycaemia" OR "postprandial glycaemia" OR "serum glucose" OR "serum sugar" OR "glucose variability" OR "glycemic variability" OR "glycaemic variability" OR "glucose control" OR "glycemic control" OR "glycaemic control" OR "diabetes control" OR "glucose tolerance" OR "diabetes remission" OR "glycated hb" in Title Abstract Keyword - in Trials

Supplementary tables

***Table S1: Relevant outcomes of included studies***

| First author (year) | Glycemic outcomes | Insulin sensitivity | T2D remission | Diabetes medication | Quality of life |
| --- | --- | --- | --- | --- | --- |
| Athinarayanan (2019) | HbA1c, fasting glucose | Fasting insulin, C-peptide, HOMA-IR, HOMA-B, HOMA-S | Prevalence and resolution of T2D (diabetes reversal, partial and complete remission) | Change in overall diabetes medication use, use by class and insulin dose | NR |
| Brown (2020) | HbA1c, fasting glucose, postprandial glucose following MMTT | Fasting plasma C-peptide, hormonal responses during the MMTT | NR | Change in number taking insulin and insulin dose | EuroQol-5 Dimension |
| Daly (2006) | HbA1c | NR | NR | Post-study analysis only assessed change in medication | NR |
| Davis (2009) | HbA1c | NR | NR | Reported descriptively in results but not included as pre-specified outcome | NR |
| Goday (2016) | Hba1c, fasting glucose | Insulin, HOMA-IR | NR | Patients treated with oral antidiabetic drugs | Patient satisfaction was assessed by the Liker Scale |
| Guldbrand (2012) | HbA1c | NR | NR | Insulin, metformin, glibenclamide dose | NR |
| Gulsin (2020) | HbA1c, fasting glucose | Fasting insulin, HOMA-IR | Diabetes remission | NR | NR |
| Iqbal (2010) | HbA1c, fasting glucose | NR | NR | NR | NR |
| Lean (2019) | HbA1c | NR | Diabetes remission | Exploratory analyses: Number of participants taking prescribed oral antidiabetic medications; number taking 0, 1 or ≥2 oral antidiabetic medications; mean number of prescribed oral antidiabetic medications | EuroQol 5 Dimension |
| Morris (2020) | HbA1c, fasting glucose | Fasting insulin, HOMA-IR, HOMA-B, HOMA-S | Change in diabetes status reported as an exploratory outcome | Change in diabetic medications | Questionnaires assessed participants’ quality of life (using PAID scale), as well as self-reported motivation and perceptions across domains of diet, health and diabetes control |
| Sato (2017) | HbA1c | NR | NR | Reduction of diabetes medications; change in insulin dosage | NR |
| Taheri (2020) | HbA1c, fasting glucose, glycemic variability (via CGM) | HOMA-IR, Quantitative insulin sensitivity check index (QUICKI) | Diabetes remission | Number of participants taking prescribed oral antidiabetic medications; number taking 0, 1 or ≥2 oral antidiabetic medications; mean number of prescribed oral antidiabetic medications | EuroQol 5 Dimension, Hospital Anxiety and Depression Scale |
| Tay (2018) | HbA1c, glycemic variability (via CGM) | Insulin, HOMA-IR, HOMA-B | NR | Antiglycemic medication effects score [MES] (based on potency and dosage of antiglycemic agents and insulin usage) | NR |
| Westman (2008) | HbA1c, fasting glucose | Fasting insulin | NR | Reduction or elimination of diabetes medications | NR |
| Yamada (2014) | HbA1c, fasting glucose | NR | NR | NR | Diabetes Treatment Satisfaction Questionnaire (DTSQ) and the Problem Areas In Diabetes (PAID) scale |

T2D, type 2 diabetes; HbA1c, glycosylated haemoglobin; HOMA-IR, Homeostatic Model Assessment for Insulin Resistance; HOMA-B, Homeostatic Model Assessment for Beta-cell function; HOMA-S, Homeostatic Model Assessment for Insulin Sensitivity; MMTT, Mixed Meal Tolerance Test; PAID, Problem Areas In Diabetes; CGM, continuous glucose monitoring; QUICKI, Quantitative insulin sensitivity check index; MES, medication effects score; DTSQ, Diabetes Treatment Satisfaction Questionnaire

***Table S2: Baseline participant characteristics of intervention arm***

| First author (year) | Mean age in years (SD) | Gender (% female) | Ethnicity | Mean BMI (SD) | Mean duration of known T2D in years | T2D medication |
| --- | --- | --- | --- | --- | --- | --- |
| Athinarayanan (2019) | 54 (8) | 67 | 6.87% African American | 40.4 (8.8) | 8.44 | 88% prescribed T2D medication (57% were prescribed a diabetes medication other than metformin, 30% prescribed insulin) |
| Brown (2020) | 58.5 (50.1-64.2) [median, IQR] | 55.6 | 57.8% Caucasian, 2.2% Mixed, 8.9% Asian, 1.1% Black | 36.6 (5.1) | 13 | 100% taking insulin, median duration 4 (2.0-6.2) years; other medications detailed |
| Daly (2006) | 58.2 (1.6) | 51 | NR | 35.4 (0.7) | NR | 40% oral antiglycemic medication, 20% insulin, 20% both |
| Davis (2009) | 54 (6) | 82 | 62% Black, 15% Hispanic, 15% White, 4% Asian, 4% Other | 35 (6) | NR | 78% metformin, 44% sulfonylurea, 35% insulin |
| Goday (2016) | 54.89 (8.81) | 67 | NR | 33.25 (1.52) | NR | 73.3% on oral antidiabetic medication |
| Guldbrand (2012) | 61.2 (9.5) | 53 | NR | 31 (4.5) | 9.8 | 50% on oral antiglycemic medication; 33% oral antiglycemic + insulin |
| Gulsin (2020) | 49.7 (6.3) | 41 | 55% Caucasian, 45% Black or ethnic minority | 37.2 (6.1) | 4.7 | 82% metformin, 13% sulfonylurea, 17% DPP-4 inhibitor, 10% SGLT2 inhibitor, 10% GLP-1 receptor agonist |
| Iqbal (2010) | 60 (8.9) | 16 | 34.4% White, 62.9 African American, 1.4% Latino, 1.4% Other | 38.1 (5.5) | NR | 84.3% oral antiglycemic medication; 22.9% insulin |
| Lean (2019) | 52.9 | 44 | 98% White | 35.1 (4.5) | 3 | 74.5% oral antiglycemic medication (44% taking 1, 31% taking ≥2); 0% insulin |
| Morris (2020) | 69 (10) | 43 | 100% White | 34.8 (3.4) | 9.0 | Average no. of diabetes medication was 1.4 |
| Sato (2017) | 60.5 (10.5) | 23 | NR | 26.7 (25-30) | 14 | 23% intensive insulin therapy; 63% metformin; others detailed |
| Taheri (2020) | 41.9 (5.4) | 30 | 100% Middle East and North Africa region | 35 (5.2) | 1.8 | Average no. of diabetes medication was 1.52; 87% metformin; 1% insulin; others details |
| Tay (2018) | 58 (7) | 36 | NR | 34.3 (4.5) | 7 | 79% metformin; 10% insulin |
| Westman (2008) | 51.8 (7.3) | 76 | 57.9% White, 36.8% African-American | 37.7 (6.1) | NR | 95.2% taking antiglycemic medications (insulin + oral agents 25%, insulin only 20%, oral agents only 60%) |
| Yamada (2014) | 63.3 (11.7) | 42 | NR | 24.5 (4.3) | 8.9 | 100% taking antiglycemic medications; 25% insulin |

SD, standard deviation; T2D, type 2 diabetes; IQR, interquartile range.

***Table S3: Intervention details***

| First author (year) | Programme | Setting | Stages (if more than one) | Key dietary advice provided | Intervention approach/intensity | Behavioural support | Physical activity |
| --- | --- | --- | --- | --- | --- | --- | --- |
| Athinarayanan (2019) | Virta Health | App-based with or without onsite-clinic support | NA | CHO level adjusted to reach ketosis. Daily protein intake 1.5 g kg-1 of ideal body weight. Incorporate dietary fats to satiety. Consume adequate omega-3 and omega-6; remainder of fat intake from both monounsaturated and saturated sources. Other aspects of the diet were individualised. | Access to web app for individual communication with care team, online resources, biomarker tracking and online peer community for social support. Education provided through app or clinic-based group meetings (weekly for 12 weeks, bi-weekly for 12 weeks, monthly for 6 months, and then quarterly in the second year). | Behaviour change strategies involving range of techniques individualised to each ppt for maintenance of lifestyle changes. | NR |
| Brown (2020) | Cambridge Weight Plan | Secondary care outpatient | Three stages: 12 week TDR; 2-12 weeks structured food reintroduction; ongoing follow up with energy deficit diet | NR | 1:1 30-60min session with dietician after 1 week and then monthly for the first 6 months (8x), plus seven 15-20min telephone consultations. Two 1:1 sessions from 6 to 12 months. | Participants received behavioural support to aid lifestyle adherence and maintenance. | Encouraged to undertake moderate exercise of at least 30 minutes, 5 days per week including both aerobic and resistance exercise |
| Daly (2006) | NA | Hospital outpatient | NA | Emphasis on incorporating at least 1/2 pint of milk and 1 piece of fruit into daily 70g carbohydrate allowance to improve vitamin/mineral intake. | One 1:1 consultation, 3-monthly group sessions and final assessment consultation. | NR | Advice on increasing physical activity incorporated into the 3 education sessions. |
| Davis (2009) | Modelled after the Atkins-diet | Research centre | NA | NR | 1:1 sessions 1 to 2 times/week for first month; then every 6 weeks. Totalled 6x 30min visits over 12 months. | NR | Recommendations to achieve 150 minutes each week but stated that physical activity not emphasis of study. |
| Goday (2016) | DiaproKal Method | Hospital outpatient | Three stages: active (very low calorie ketogenic phase, maintained for 30 to 45 days until 90% of target weight lost); metabolic stabilisation phase (low calorie); maintenance (balanced diet). | Meal replacements based on a high-biological-value protein preparations diet and natural foods. Consume fat rich in MUFA and protein from poultry and fish rather than from saturated fat-rich red meat. | 1:1 session and telephone contact/every 15 days. Totalled 9 times in 4 months. | Counselling to support lifestyle and behavioural modification. Details NR. | NR |
| Guldbrand (2012) | NA | Primary care | NA | NR | Group sessions 4 times over 12 months. | NA | No information given to change level of activity. |
| Gulsin (2020) | Cambridge Weight Plan | Research centre | Two stages: TDR, which was continued until 12 weeks or once 50% EBW had been lost, whichever came first; followed by maintenance diet. | NR | 1:1 weekly contact with dietician or physician. | Health behaviour coaching and relapse prevention | Advised to maintain usual daily activities. |
| Iqbal (2010) | NA | Web app-based with or without onsite-clinic support | NA | Select wholegrain products and foods with high fibre content. Don't restrict total fat or caloric intake but consume 'healthy fats' (MUFA and PUFA) and minimise intake of saturated and trans fats. | Weekly 2-hour group nutrition education classes for the first month with opportunity to speak to dietician at end. Thereafter, monthly group sessions for 11 months. | NR | Encouraged to engage in at least 30 minutes of moderate activity at least 5 times/week. |
| Lean (2019) | Cambridge Weight Plan | Hospital outpatient | Three stages: TDR (825–853 kcal/day formula diet for 3–5 months); stepped food reintroduction (2–8 weeks); and structured support for long-term weight loss maintenance. | Follow Eatwell guidelines during phases 2 and 3, although dietary advice may be individualised. | 1:1 sessions every 2 weeks in phases 1 and 2; and monthly in phase 3. Total of 35x over 24 months. | Behavioural change methods incorporated in the weight loss maintenance phase, including elements of cognitive behavioural therapy. | Encouraged to maintain usual physical activities during TDR. In weight loss maintenance phases, step-counters provided with the recommendation to reach and maintain individual sustainable maximum, up to 15,000 steps/day. |
| Morris (2020) | DIAMOND programme | Primary care | Two stages: 8 week low carbohydrate LED; 4 weeks gradually increasing energy intake by increasing portion size at one meal or adding one portion of high fibre CHO. | Exclude sugary and starchy foods high in CHO entirely from the diet (with the exception of dairy and limited fruit intake), strict portion control and minimal use of fats and oils. Eat fresh vegetables or salad and small amounts of lean meat and fish. | 1:1 session with practice nurses at baseline, weeks 2, 4 and 8. | Advice on goal-setting, self-monitoring and problem-solving strategies. | NR |
| Sato (2017) | NA | Research centre | NA | At each meal, consume about 23.3 g CHO. Consume unsaturated fat rather than saturated fat. | 1:1 sessions for 30 minutes at 0, 1, 2, 4 and 6 months. | NR | NR |
| Taheri (2020) | Cambridge Weight Plan | Research centre | Three stages: TDR (825–853 kcal/day formula diet for 3–5 months); stepped food reintroduction (2–8 weeks); and structured support for long-term weight loss maintenance. | Eating raw vegetables and salad was permitted in the TDR phase, if required. Drink 2L or more of water daily. | 1:1 sessions every 2 weeks in phases 1 and 2; and monthly in phase 3. Total of 35x over 24 months. | Behavioural change methods incorporated in the weight loss maintenance phase, including elements of cognitive behavioural therapy. | Physical activity support initially focused on walking (with an aim of at least 10 000 steps per day), followed by the recommendation of increasing unsupervised activity to at least 150 min/week. Participants were provided with a wrist-worn accelerometer and were directed to smartphone apps to monitor food intake and activity. |
| Tay (2018) | NA | Hospital outpatient | NA | 30 g high-fibre, low GI cereal; 1 crispbread; 250 g lean chicken, pork, fish, red meat (3 to 4 times/week); 40 g almonds and 20 g pecans; 3 cups low-starch vegetables; 200 mL skim (<1% fat) milk; 100 g diet yogurt; 20 g cheese; 30 g margarine/oil. | 1:1 sessions every 2 weeks for 12 weeks and monthly thereafter. | NR | Circuit training 3 days/week with moderate intensity aerobic/resistance exercises. |
| Westman (2008) | NA | Primary care | NA | Unlimited amounts of meat, fish and eggs; limited amounts hard cheese (4 oz/day), fresh cheese (eg, cottage/ricotta, 2 oz/day), salad vegetables (2 cups/day), and non-starchy vegetables (1 cup/day). Drink at least 6 glasses of permitted fluids daily. | Group sessions every week for 3 months, then every other week for 3 months. | NR | Encouraged to exercise for 30 minutes at least 3 times/week. |
| Yamada (2014) | NA | Research centre | NA | Target CHO content in each meal was 20-40 g, allowed to consume sweets containing 5g of CHO twice daily. | 1:1 session every 2 months. | NR | NR |

NA, not applicable; CHO, carbohydrate; NR, not reported; TDR, total diet replacement; DIAMOND, DIetary Approaches to the Management Of type 2 Diabetes; MUFA, monounsaturated fatty acids; PUFA, polyunsaturated fatty acids; LED, low energy diet.

***Table S4: Assessment of adherence, prescribed and reported carbohydrate intakes***

| Study | Assessment of adherence | Prescribed CHO (g) | Reported CHO (g) |
| --- | --- | --- | --- |
| Athinarayanan (2019) | BHB concentrations | <30 | NR |
| Brown (2020) | NR | 115 | NR |
| Daly (2006) | 5-day food diary | <70 | **110** |
| Davis (2009) | 24-hour recall by interview, food diaries | 20-25 (increase by 5g/week) | **137** |
| Goday (2016) | Eating Self-Efficacy Scale, ketone urine sticks | <50 | NR |
| Guldbrand (2012) | 3-day food diary | 85 | **97** |
| Gulsin (2020) | NR | 101 | NR |
| Iqbal (2010) | 24-hour recall | 30 | **193** |
| Lean (2019) | NR | 124 | NR |
| Morris (2020) | Questionnaires to assess dietary intake and self-reported adherence | <59 | NR |
| Sato (2017) | 3-day food diary | 130 | **149** |
| Taheri (2020) | NR | 124 | NR |
| Tay (2018) | Daily food records | <50 | **83** |
| Westman (2008) | 5-day food diary and urinary ketones | <20 | **49** |
| Yamada (2014) | 3-day food diary | 70-130 | 126 |

Bold values deviate more than 10% from the prescribed amount.

CHO, carbohydrate; BHB, beta-hydroxybutyrate; NR, not reported

***Table S5: Summary of medication changes among intervention and control groups***

| Study name | Diabetes medication | Authors’ summary of medication changes | Greater decrease in medication in intervention |
| --- | --- | --- | --- |
| Athinarayanan (2019) | Change in overall diabetes medication use, use by class and insulin dose | In intervention, use of any glycemic control medication (excluding metformin) declined (from 55.7 to 26.8%) including insulin (-62%) and sulfonylureas (-100%). No changes in diabetes medication use in control. | Yes |
| Brown (2020) | Change in number taking insulin and insulin dose | Insulin therapy was discontinued in 39.4% of the intervention group compared with 5.6% of the control group among completers. Insulin requirements fell by 47.3 units (SD 36.4) in the intervention compared with 33.3 units (SD 52.9) in the control (−18.6 units, 95% CI −29.2 to –7.9, p=0.001). | Yes |
| Daly (2006) | Post-study analysis only assessed change in medication | Patient reported data were available for only 75% of subjects. Insulin was reduced in approximately 85% of insulin using subjects in the LC group, but in only 22% of subjects in the LF group. In contrast, approximately 16% of LF subjects increased insulin compared with 5% in LC subjects. | Yes |
| Davis (2009) | Reported descriptively in results but not included as pre-specified outcome | Of the participants using insulin, the dose was reduced by a mean (SD) of 10 (14) units in the low-carbohydrate arm and increased by 4 (19) units in the low-fat arm (P= 0.12) at 12 months. The change in sulfonylurea dose was a 1.6 (3.6) mg reduction in both arms. | Yes |
| Dyson (2007) | NR - only included patients with metformin | NR | NR |
| Goday (2006) | Patients treated with oral antidiabetic drugs | More frequent medication reduction/elimination in low carbohydrate diet than the low glycemic index diet. | Yes |
| Guldbrand (2012) | Insulin, metformin, glibenclamide dose | The reduction in insulin was statistically significant only in the low-carbohydrate group at 6 months. This change in the average insulin dose was statistically significant between the two groups at 6 months (p=0.046). | Yes |
| Gulsin (2020) | NR | NR | NR |
| Iqbal (2010) | NR | Inadequate reporting of medication changes | NR |
| Lean (2018) | Exploratory analyses: Number of participants taking prescribed oral antidiabetic medications; number taking 0, 1 or ≥2 oral antidiabetic medications; mean number of prescribed oral antidiabetic medications | The mean number of antidiabetic medications prescribed decreased in the intervention group and increased in the control group. | Yes |
| Morris (2020) | Change in diabetic medications | In the intervention group, seven participants stopped one or more diabetic medications and seven stopped one or more hypertensive medications over the 12-week study period. There was no change in either class of drug in the control group. The adjusted difference in the number of diabetes medications was −0.4 (−0.8 to −0.001, P = 0.051). | Yes |
| Sato (2017) | Reduction of diabetes medications; change in insulin dosage | Six patients of the LCD group were able to reduce the glucose-lowering medications through the intervention. Only 1 was able to reduce glucose-lowering medications in the calorie-restricted group. | Yes |
| Taheri (2020) | Number of participants taking prescribed oral antidiabetic medications; number taking 0, 1 or ≥2 oral antidiabetic medications; mean number of prescribed oral antidiabetic medications | In the intervention group, improvements in HbA1c were accompanied by fewer participants taking diabetes medications (four [6%] of 68 participants) compared with the control group (58 [81%] of 72 participants). | Yes |
| Tay (2018) | Antiglycemic medication effects score [MES] (based on potency and dosage of antiglycemic agents and insulin usage) | Over twice the number of LC participants had a ≥ 20% reduction in MES compared to HC participants (LC, 22; HC, 9). | Yes |
| Westman (2008) | Reduction or elimination of diabetes medications | Diabetes medications were reduced or eliminated in 95.2% of LCKD vs. 62% of LGID participants (p < 0.01). | Yes |
| Yamada (2014) | NR | NR | NR |

LC, low carbohydrate; LF, low fat; NR, not reported; LCD, low carbohydrate diet; MES, medication effects score; HC, high carbohydrate; LCKD, low carbohydrate ketogenic diet; LGID, low glycemic index diet.

***Table S6: Association between average weight loss and change in HbA1c at 3, 6, 12 and 24 months***

Correlation analysis was conducted to compute the Pearson correlation coefficient for the association between average weight loss and HbA1c change across studies at specific time-points. This was tested for significance using a t-test.

| **Duration (months)** | **Number of studies (N)** | **Correlation** | **p-value** | **R-squared** |
| --- | --- | --- | --- | --- |
| 3 | 6 | No | 0.673 | 0.05 |
| 6 | 7 | Yes | 0.004 | 0.84 |
| 12 | 9 | Yes | 0.001 | 0.82 |
| 24 | 5 | Yes | 0.010 | 0.92 |

## Supplementary figures

***Figure S1: Average improvement in HbA1c and average percentage weight loss at 24 months.***

Each point represents the mean value for a single study. Squares, no energy restriction (*ad libitum* feeding); circles, moderate energy restriction (1200-2000kcal/day); triangles, severe energy restriction (<1200kcal/day).
